# Role of balloon dilation test in identifying suitable candidates for gastric peroral endoscopic myotomy

**DOI:** 10.1002/deo2.70049

**Published:** 2025-01-15

**Authors:** Boldbaatar Gantuya, Haruhiro Inoue, Kazuki Yamamoto, Miyuki Iwasaki, Kei Ushikubo, Yohei Nishikawa, Hidenori Tanaka, Ippei Tanaka, Mayo Tanabe, Satoshi Abiko, Yuto Shimamura

**Affiliations:** ^1^ Digestive Diseases Center Showa University Koto Toyosu Hospital Tokyo Japan; ^2^ Department of Gastroenterology Mongolian National University of Medical Sciences Ulaanbaatar Mongolia; ^3^ Endoscopy Unit Mongolia Japan Hospital Ulaanbaatar Mongolia

**Keywords:** balloon dilation, gastric peroral endoscopic myotomy, gastroparesis

## Abstract

**Background:**

Predicting successful outcomes of gastric peroral endoscopic myotomy (G‐POEM) is essential for identifying patients who are most likely to benefit from the procedure. This study aimed to evaluate the utility of the balloon dilation test (BDT) in optimizing patient selection for G‐POEM.

**Methods:**

A retrospective study was conducted from February 2021 to December 2023, including patients with refractory gastroparesis unresponsive to conventional medical treatments. All patients completed the Gastroparesis Cardinal Symptom Index (GCSI) questionnaire before and after G‐POEM. The pyloric ring balloon dilation test was performed prior to G‐POEM, with only patients who showed a positive response included. Procedural and clinical outcomes were analyzed.

**Results:**

Five patients (three female and two male) with refractory gastroparesis were included. The median G‐POEM procedure time was 60 minutes (interquartile range [IQR] 32.5–110), and technical success was achieved in all cases. At a median follow‐up of 10 months (IQR 6–34), clinical response was observed in all patients (100%). The GCSI score improved significantly from a median of 17 (IQR 8–33) to 0 (IQR 0–4.5; *p* < 0.03) indicating significant improvement in clinical symptoms.

**Conclusion:**

A positive response to the balloon dilation test appears to be a reliable predictor of successful short‐term outcomes following G‐POEM in patients with refractory gastroparesis.

## INTRODUCTION

Gastroparesis is a syndrome characterized by delayed gastric emptying of solids in the absence of mechanical obstruction, accompanied by cardinal symptoms such as nausea, vomiting, early satiety, belching, bloating, and upper abdominal pain.^1^ Idiopathic gastroparesis is the most common subtype, with approximately half of cases having no identifiable underlying abnormality. Other etiologies include diabetic, iatrogenic, and postsurgical causes,^2^ often linked to damage to the vagus nerve, interstitial cells of Cajal, smooth muscle cells, and enteric neurons.^3^, ^4^, ^5^ The pathophysiology involves multiple mechanisms, including impaired gastric accommodation, fundic dysfunctions, antroduodenal discoordination, and pylorospasm,^6^, ^7^, ^8^ all contributing to distressing symptoms that significantly impair quality of life.^9^, ^10^ Severe cases may lead to complications such as weight loss and malnutrition.^11^

Management of gastroparesis typically follows a stepwise approach, beginning with dietary modifications and medical therapies, and progressing to surgical or endoscopic interventions as needed.^12^, ^13^, ^14^, ^15^ However, approximately 30% of patients experience inadequate symptom relief with conservative approaches.^16^ Refractory gastroparesis is defined as poor symptom control after six months of dietary and pharmacological interventions. Long‐term use of prokinetic agents is limited by adverse effects, such as QTc prolongation, extrapyramidal symptoms, and tachyphylaxis.^17^ Additionally, prolonged use of metoclopramide has been associated with an increased risk of Parkinsonism in diabetic gastroparesis¹. Many patients ultimately fail to achieve sustained symptom control either due to loss of efficacy or adverse side effects.^13^, ^18^

Given the rising prevalence of gastroparesis and the limitations of existing therapies, alternative strategies are increasingly sought.^16^ These alternatives include gastric electrical stimulation, botulinum toxin injections, and transpyloric stenting, though these are not widely adopted due to their invasiveness and inconsistent outcomes.^11^ Surgical options, although available, are less favorable due to their invasive nature and relatively low success rates.^19^

Based on the successful outcomes of per‐oral endoscopic myotomy (POEM) for esophageal achalasia, gastric POEM (G‐POEM) has been introduced as a treatment for gastroparesis. First performed successfully by Inoue and Khashab,^20^ G‐POEM has gained recognition as a minimally invasive option targeting pyloric dysfunction. Studies using endoscopic functional luminal imaging probes (EndoFLIP) indicate that approximately 45% of patients with gastroparesis exhibit high‐amplitude pyloric contractions,^21^ supporting the use of endoscopic therapies focused on the pylorus.^22^, ^23^, ^24^, ^25^ However, clinical success rates of G‐POEM range from 50% to 80% in the short term (6 months to 1 year).^11^

Identifying predictors of G‐POEM success is essential to improve patient selection. Factors such as high body mass index, long‐standing gastroparesis, and the use of psychiatric or narcotic medications have been associated with poorer outcomes. A recent international prospective trial suggests that a baseline Gastroparesis Cardinal Symptom Index (GCSI) score above 2.6 and gastric retention exceeding 20% at 4 h on scintigraphy predict favorable outcomes at 1 year.^26^ Other characteristics, such as the presence of bile in the stomach, absence of pylorospasm, and tight pylorospasm, have not shown predictive value.^27^ Endoscopic findings have yet to reliably predict outcomes, and predictors of clinical success remain uncertain.^11^ Unlike the esophagus, evaluating all parts of the gastric motility is limited by its anatomical feature. Although gastric emptying scintigraphy is commonly used, its utility is hindered by variability in test performance and the lack of standardized normal values, reducing its routine application. Balloon dilation has demonstrated efficacy in alleviating pyloric stenosis or spasms, though its long‐term effectiveness is limited. This study investigates the utility of balloon dilation testing (BDT) prior to G‐POEM as a predictor of clinical success, aiming to enhance patient selection and achieve sustained treatment outcomes in refractory gastroparesis.

## METHODS

We conducted a retrospective study from February 2021 to December 2023, including consecutive patients with gastroparesis who had not responded to multiple medical treatments. All patients underwent diagnostic evaluations for gastroparesis, which included barium studies, esophagogastroduodenoscopy (EGD), and the GCSI questionnaire.^28^ Exclusion criteria included a history of surgical gastrectomy, the presence of space‐occupying lesions or luminal narrowing at the gastric outlet, and cases where G‐POEM was performed without a prior BDT or following a failed BDT. Clinical response was defined as a reduction of >25% in at least two GCSI cardinal symptom subscales. Recurrence was defined as either a return to baseline GCSI scores or an increase of > 3 points in GCSI scores, sustained for at least three months after an initial clinical response.^17^ The study protocol was approved by the Ethics Committee of Showa University (IRB number 2024‐147‐A) and was conducted in accordance with the 2013 revision of the Declaration of Helsinki. As this was a retrospective study, individual informed consent was waived; instead, study details were disclosed on the hospital website, allowing patients the opportunity to opt‐out.

### Balloon dilation test techniques

**FIGURE 1:**
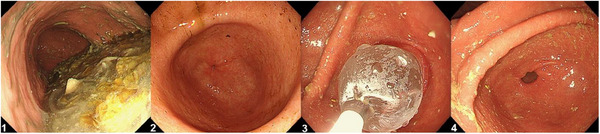
Gastric balloon dilation test. 1. Food residue in the stomach, indicative of gastroparesis. 2. Gastric antrum prior to dilation, with no obvious pyloric obstruction. 3. Balloon dilation of the pyloric sphincter using a 20–21  mm dilator. 4. Post‐dilation showing visible pyloric opening.

All patients underwent a BDT as a prerequisite to G‐POEM. The procedure utilized either the EZDilate 20‐mm Endoscopic Balloon Dilator (Olympus Corporation, Tokyo, Japan) or the Elation5 21‐mm Dilation Balloon (Merit Medical, South Jordan, Utah, USA). The balloon was inflated twice, each for one minute, under sedation, with the patient positioned in the left lateral decubitus position (Figure 1).

The effectiveness of the BDT was evaluated within one month using the GCSI questionnaire. A positive response to the BDT was defined as a > 25% reduction in at least two GCSI symptom subscales. Patients demonstrating a clinical response proceeded to undergo G‐POEM, whereas those without significant improvement did not undergo the procedure.

### G‐POEM techniques

Patients were placed on a liquid diet the day prior to the procedure and fasted overnight. G‐POEM was performed with the patient in the left lateral position under general anesthesia. The procedure utilized the Triangle Tip Jet Knife (Olympus) and either the GIF‐H290T or GIF‐Q260J gastroscope (Olympus), both equipped with water jet functions. To enhance visualization and maneuverability, a SpaceAdjuster (Top Corporation, Tokyo, Japan) or a transparent hood (ST Hood; Fujifilm, Tokyo, Japan) was attached to the gastroscope tip, and carbon dioxide was used for insufflation throughout the procedure.

**FIGURE 2:**
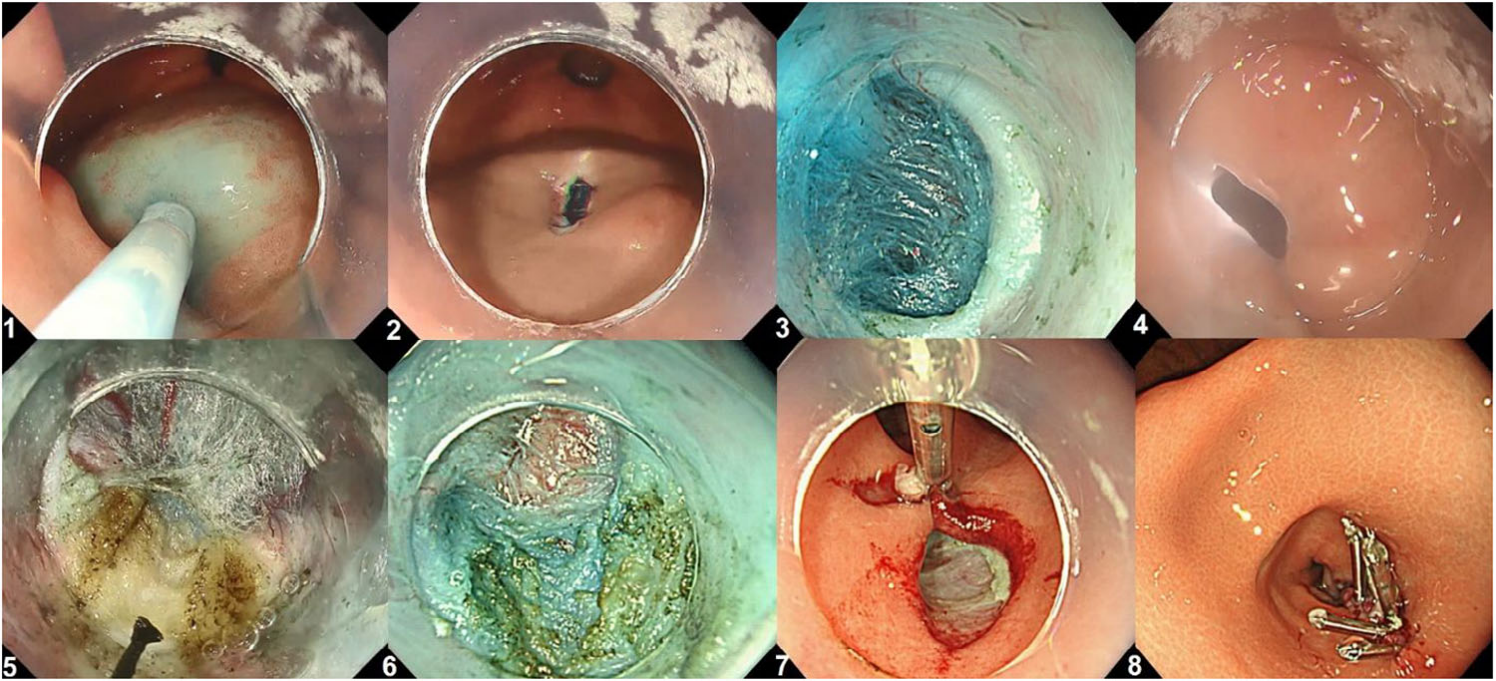
Procedural steps of gastric peroral endoscopic myotomy. 1. Submucosal injection with saline and indigo carmine. 2. Mucosal incision to access the submucosal space. 3. Creation of a submucosal tunnel toward the pylorus. 4. Distal endpoint of the tunnel visualized with transillumination with double‐scope method. 5. Myotomy of the pyloric circular muscle. 6. Completion of the myotomy. 7. Closure of the mucosal entry site using hemostatic clips. 8. Restoration of gastric wall integrity.

The procedure began with an injection of saline mixed with indigocarmine dye approximately 5 cm proximal to the pylorus. A 2‐cm mucosotomy was created at the greater curvature of the antrum using a VIO300D or VIOD3 electrosurgical generator (ERBE Elektromedizin, Tubingen, Germany) to access the submucosal space. Submucosal tunneling extended from the mucosotomy to 1 cm distal to the pylorus, reaching into the duodenal bulb. A pyloromyotomy was then performed using the Triangle Tip Jet Knife. Electrosurgical settings were as follows: VIO300D: ENDOCUT I (Effect 2, Duration 1, Interval 6 on VIO300D; Effect 2, Duration 2, Interval 2 on VIO3) for mucosotomy, and SPRAY COAG (Effect 2, 50 W on VIO300D; Effect 4.0 on VIO3) for submucosal tunneling. The mucosal entry was closed using endoscopic clips (Figure 2).

### Postoperative management

To prevent infection‐related complications, first‐generation cephalosporins were administered for three days postoperatively, and patients received proton pump inhibitors (20 mg daily) for 8 weeks. Patients without signs of abdominal pain or peritonitis were allowed to begin drinking clear fluids on the day after the procedure. A liquid diet was introduced on postoperative day two, with progression to a regular diet by day three. Symptom severity was reassessed using the GCSI questionnaire after G‐POEM, with follow‐up evaluations conducted six months post‐procedure.

### Statistical analysis

Continuous variables were expressed as median (interquartile range, IQR), while categorical variables were reported as frequencies and percentages. For paired data, the efficacy of G‐POEM was analyzed using the Wilcoxon signed‐rank test. All statistical analyses were performed using JMP Version 17.0 (SAS Institute Inc.), with a *p*‐value of < 0.05 considered statistically significant.

## RESULTS

**FIGURE 3:**
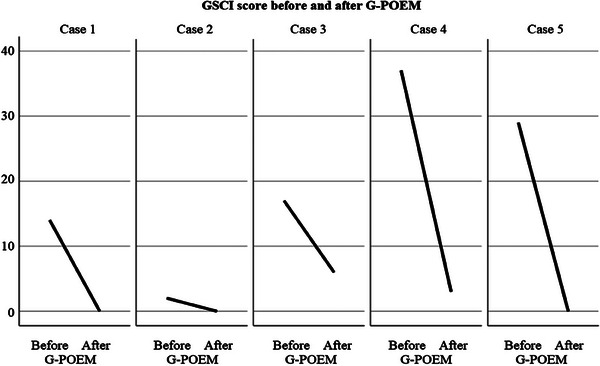
Comparison of the Gastroparesis Cardinal Symptom Index (GCSI) total scores before and after gastric peroral endoscopic myotomy (G‐POEM) for all cases.

**FIGURE 4:**
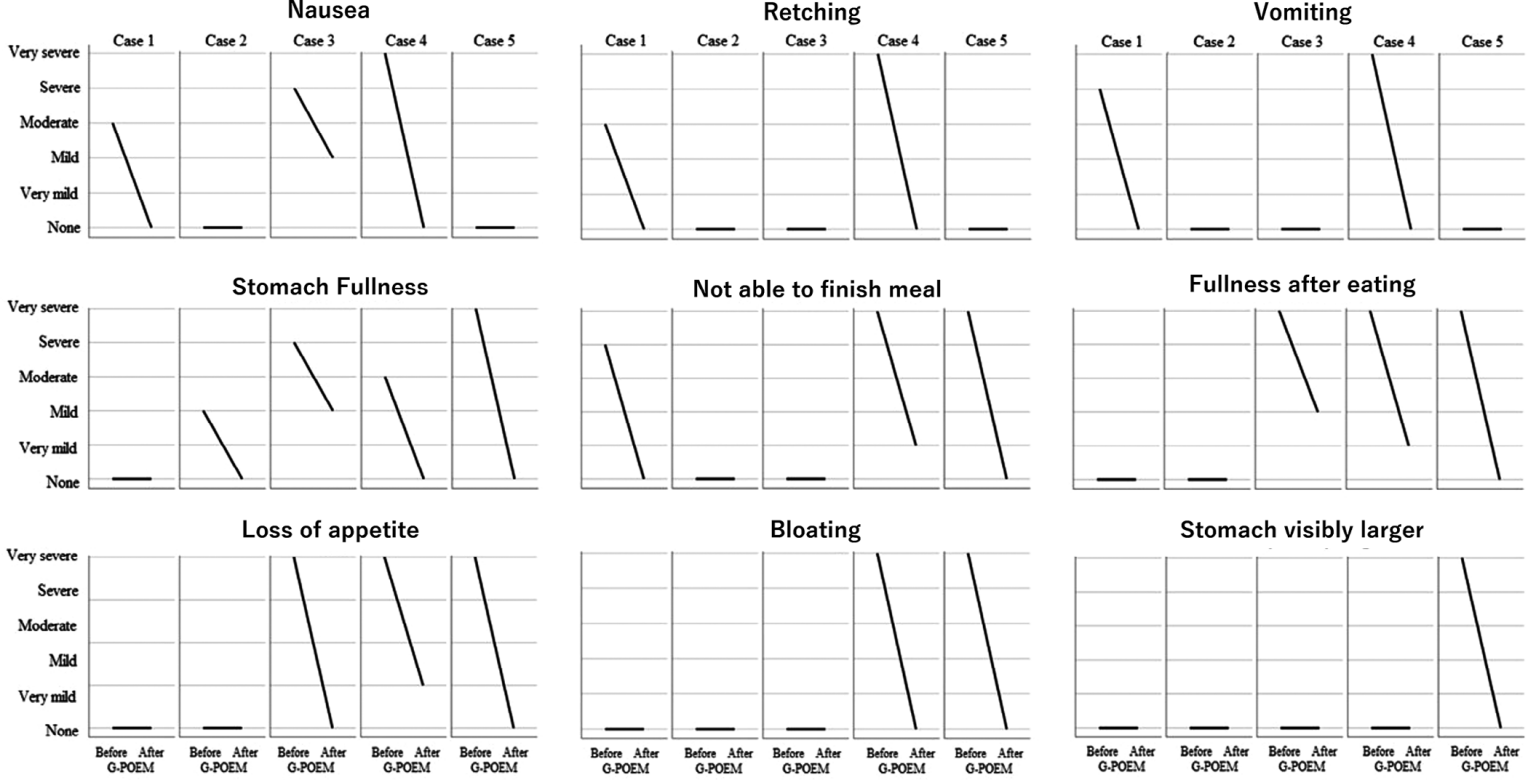
The Gastroparesis Cardinal Symptom Index (GCSI) scores before and after gastric peroral endoscopic myotomy (G‐POEM), illustrating individual patient outcomes.

A total of five patients (three female and two male) with refractory gastroparesis who demonstrated a positive response to the BDT were included in the study. The median age of the cohort was 60 years (IQR 47.5–75). The median procedure duration was 60 minutes (IQR 32.5–110; Table 1), and the median hospital stay was 3 days. Technical success was achieved in all cases, with no intraoperative complications reported. At a median follow‐up of 10 months (IQR 6–34), clinical response was observed in all patients (100%). Post‐procedure, clinical symptoms improved significantly compared to the pre‐procedure status. The GCSI score decreased significantly from a median of 17 (IQR 8–33) to 0 (IQR 0–4.5; *p* < 0.03) following G‐POEM. Demographic data and clinical outcomes of G‐POEM are detailed in Table 1. Individual patient GCSI scores before and after G‐POEM are illustrated in Figure 3, and case‐specific changes in GCSI cardinal symptoms are shown in Figure 4. During the follow‐up period, no patients experienced a recurrence of clinical symptoms. Furthermore, no major complications or mortality were reported.

**TABLE 1:** Demographic data and clinical outcome of gastric peroral endoscopic myotomy (G‐POEM).

	Case 1	Case 2	Case 3	Case 4	Case 5	Median	IQR
Gender	Female	Male	Male	Female	Female	n/a	n/a
Age	72	60	42	78	53	60	47.5–75
Procedure time (min)	60	39	26	120	100	60	32.5–110
Balloon diameter (mm)	21	20	20	20	20	20	20–20.5
Etiology of gastroparesis	Post Heller Dor	Post ablation for Afib	Idiopathic	Post ablation for Afib	Idiopathic	n/a	n/a
Previous interventions	None	None	None	None	None	n/a	n/a
Comorbidity	Achalasia	None	Reflux esophagitis	Afib	None	n/a	n/a
GSCI before G‐POEM	14	2	17	37	29	17	8–33
GSCI after G‐POEM	0	0	6	3	0	0	0–4.5
Follow‐up period (months)	6	6	10	28	40	10	6–34
Before G‐POEM							
Nausea	3	0	4	5	0	3	0–4.5
Retching	3	0	0	5	0	0	0–4
Vomiting	4	0	0	5	0	0	0–4.5
Stomach fullness	0	2	4	3	5	3	1–4.5
Not able to finish the meal	4	0	0	5	5	4	0–5
Fullness after meals	0	0	5	5	5	5	0–5
Loss of appetite	0	0	4	4	4	4	0–4
Bloating	0	0	0	5	5	0	0–5
Stomach visibly larger	0	0	0	0	5	0	0–2.5
After G‐POEM							
Nausea	0	0	2	0	0	0	0–1
Retching	0	0	0	0	0	0	0–0
Vomiting	0	0	0	0	0	0	0–0
Stomach fullness	0	0	2	0	0	0	0–1
Not able to finish the meal	0	0	0	1	0	0	0–0.5
Fullness after meals	0	0	2	1	0	0	0–1.5
Loss of appetite	0	0	0	1	0	0	0–0.5
Bloating	0	0	0	0	0	0	0–0
Stomach visibly larger	0	0	0	0	0	0	0–0

Abbreviations: A‐fib; Atrial fibrillation, GSCI; Gastroparesis Cardinal Symptom Index, G‐POEM; Gastric per‐oral endoscopic myotomy, H‐D; Heller‐Dor.

## DISCUSSION

Our study demonstrated the potential role of the BDT in predicting the efficacy of G‐POEM in treating gastroparesis. We propose that the BDT can effectively identify patients most likely to benefit from G‐POEM. While previous literature reports G‐POEM success rates ranging from 47% to 83%,^11^, ^20^, ^22^, ^23^, ^24^, ^25^, ^29^ incorporating the BDT in our study resulted in a significantly higher success rate of 100% with no recurrence. The BDT temporarily dilates the pyloric muscle, simulating the therapeutic effects of pyloromyotomy during G‐POEM. Based on this, we introduce the concept of “BDT‐responsive gastroparesis” to define a subset of patients likely to achieve optimal outcomes with G‐POEM. This finding suggests that BDT may serve as a valuable pre‐procedure screening tool, improving patient selection and enhancing clinical outcomes.

EndoFLIP studies estimate that 45%‐54% of gastroparesis patients exhibit pylorospasm, with pyloric distensibility significantly reduced compared to healthy controls (16.2 vs. 25.2 mm^2^/mmHg, *p* < 0.05).^21^, ^30^ As a result, pylorus‐targeted therapies such as botulinum toxin injections, transpyloric stenting, and pneumatic balloon dilation have been developed.^3^, ^31^, ^32^, ^33^, ^34^ However, these treatments often provide only short‐term relief. For example, botulinum toxin injection has shown symptom improvement rates of 64% in 1 month, but randomized trials have found no significant long‐term benefit compared to a placebo.^32^, ^33^, ^34^ Additionally, complications such as stent migration and short‐lived effects of these treatments limit their utility.^11^

“Third‐space” endoscopic techniques, including POEM for achalasia and Zenker's diverticulum, have revolutionized the management of gastrointestinal disorders.^35^, ^36^, ^37^ G‐POEM, first performed in humans by Inoue and Khashab in 2013,^20^ has since demonstrated symptom improvement rates of 61%‐86% and normalization of gastric emptying scintigraphy in 47%–83% of patients in multicenter studies.^20^, ^22^, ^23^, ^24^, ^25^, ^29^ Compared to other treatment modalities, G‐POEM has shown superior results, with studies reporting significantly higher two‐year clinical response rates (77% vs. 54%) and lower adverse event rates (4% vs. 26%) than gastric electrical stimulation^38^, ^39^ A meta‐analysis comparing G‐POEM and surgical pyloroplasty found similar clinical success rates in terms of GCSI score (75 .8% vs. 77.3%, *p* = 0.81) and gastric emptying results (85 .1% vs. 84.0%, *p* = 0.91), with comparable adverse event rates.^23^

Although short‐term G‐POEM success rates are reported at 50%–80%, long‐term outcomes demonstrate durable efficacy for up to four years, albeit with recurrence rates of 13% or higher. Identifying predictors for G‐POEM success remains a challenge^11^ While diagnostic tools such as manometry and EndoFLIP show promise, their clinical utility requires further validation.^21^, ^31^, ^40^ Some studies have suggested potential predictors, including female gender and diabetes mellitus as negative predictors, while idiopathic etiology, prior botulinum injections, and the use of electrical stimulators have been associated with positive outcomes.^23^, ^41^ Given that G‐POEM specifically targets the pylorus, confirming pylorospasm in gastroparesis patients is critical for achieving favorable outcomes. Well‐designed prospective studies are required to evaluate pyloric distensibility before G‐POEM and identify clinical predictors of success.^42^

Our study has several limitations. First, the small sample size is a significant limitation, reducing the statistical power and generalizability of the findings. The absence of long‐term follow‐up further limited the assessment of the sustainability of the outcomes. Moreover, incorporating standardized diagnostic methods, such as gastric emptying scintigraphy, in future studies would provide a more objective assessment of gastric motility and improve the reliability of the results. A comparative study with a non‐BDT group could also offer valuable insights, helping to further validate our hypothesis and better understand the relative benefits of the treatment. While the non‐invasive GCSI score provided useful preliminary insights in this pilot study, future studies should adopt more comprehensive evaluation methods to strengthen the conclusions.

In conclusion, the BDT shows promise as a predictive tool for identifying patients with gastroparesis who are likely to benefit from G‐POEM. A positive BDT result may serve as a reliable indicator of favorable clinical outcomes following G‐POEM. Larger prospective studies incorporating advanced diagnostic methods, such as EndoFLIP and gastric emptying scintigraphy, are warranted to validate these findings and optimize patient selection criteria for this procedure.

## CONFLICT OF INTEREST STATEMENT

Haruhiro Inoue serves as an advisor for Olympus Corporation and Top Corporation. He has also received educational grants from Olympus Corporation and Takeda Pharmaceutical Co. The other authors declare no conflict of interest.

## ETHICS STATEMENT

Approval of the research protocol by an Institutional Reviewer Board: The study was approved by the Institutional Review Board of Showa University (IRB number 2024‐147‐A).

## PATIENT CONSENT STATEMENT

N/A

## CLINICAL TRIAL REGISTRATION

N/A
